# Multidimensional Pain Modulation by Acupuncture Analgesia: The Reward Effect of Acupuncture on Pain Relief

**DOI:** 10.1155/2022/3759181

**Published:** 2022-11-10

**Authors:** Sian Pan, Shaohua Wang, Xiao Xue, Hanyu Yuan, Juan Li, Yu Liu, Zenghui Yue

**Affiliations:** ^1^College of Acupuncture, Massage and Rehabilitation, Hunan University of Chinese Medicine, Changsha 410208, China; ^2^Department of Rehabilitation Medicine, Zhuzhou Central Hospital, Zhuzhou 412000, Hunan, China

## Abstract

Pain is an intrinsically unpleasant experience with features that protect an organism by promoting motivation and learning. Pain relief, a negative reinforcement of pain, is considered a reward and activates the brain's reward system. The reward circuit in the brain involves reward and pain. Acupuncture has a multidimensional and comprehensive regulating effect on chronic pain. However, the reward effect of acupuncture in relieving chronic pain and the mechanism of the brain reward circuit involved in acupuncture analgesia are not thoroughly studied. In this article, we have reviewed the definition of pain abnormalities and negative emotions in patients with chronic pain, the conceptual characteristics of analgesic reward, and the new progress in studying brain reward circuits and functions. Moreover, we have expounded on the critical clinical and scientific significance of studying the reward effect of acupuncture analgesia and related brain reward circuits, the pain mechanism obtained from human neuroimaging studies, and the survey results on the effects of acupuncture on reward/motivation circuits. Some viewpoints and suggestions on the reward effect of acupuncture analgesia and related reward circuits have been put forward to clarify the multidimensional characteristics and benign regulation of acupuncture analgesia. Studies on the reward effect of acupuncture in relieving chronic pain and the regulating effect of the brain reward loop on acupuncture analgesia help to deepen the clinical understanding of acupuncture analgesia, innovate the research concept of acupuncture analgesia, and provide help for further studies on the central mechanism of acupuncture in improving chronic pain in the future.

## 1. Introduction

Acupuncture analgesia has a long history, and its effectiveness, simplicity, and few adverse reactions have been widely recognized at national and international levels. Of the 64 indications for acupuncture recommended by the 1996 World Health Organization Conference in Milan, Italy, 32 are related to pain. Currently, pain remains one of the main indications for acupuncture. Acupuncture analgesia relieves patients' degree of pain and avoids addiction, immunosuppression, gastrointestinal injury, and other adverse reactions caused by taking analgesic drugs. It improves the quality of life of patients with pain; therefore, acupuncture analgesia has been increasingly used in clinical practice. Several reports have demonstrated the efficacy of acupuncture in treating inflammatory, neuropathic, cancer, or other types of pain. However, due to the multitargeted characteristics of the acupuncture effect, the mechanism of acupuncture analgesia has not been fully elucidated.

Previous basic and clinical studies on acupuncture analgesia have focused on reducing pain perception and incidence frequency and improving the quality of life [1]. With the advancement of studies on pain, these previous studies have found that acupuncture can ease the pain of patients and further regulate the negative emotions and cognition induced by pain. Therefore, as a new perspective of acupuncture analgesia, studies assessing the appropriate strategies on how to improve pain threshold and regulate an individual's pain-related emotion and cognition level, changing from a single mode of nociceptive feeling to a multidimensional way of “pain-emotion-cognition,” are required in the future.

## 2. Pain: Not Just a Feeling

Pain is a complex physical and psychological activity and is one of the most common clinical symptoms. Professor Campbell, former president of the American Pain Society, proposed to rank pain as the fifth vital sign after pulse, blood pressure, respiration, and body temperature [2]. As early as 1979, the International Association on the Study of Pain (IASP) and World Health Organization defined pain as “an unpleasant feeling, emotional experience, or description associated with actual or potential tissue damage” [3]. Tissue damage is a standard prerequisite for pain. However, occasionally, pain occurs when tissue damage cannot be observed. To accommodate this development, the definition of pain was revised by the IASP in 2020, redefining “pain as a distressing experience associated with actual or potential tissue damage with sensory, emotional, cognitive, and social components” [4]. The new definition emphasizes that pain emotion, cognition, and sociability are essential dimensions in evaluating pain, in addition to the feeling of hurt, which is the feeling of pain.

Pain can be divided into two categories: physiological and pathological pain. Physiological pain, also known as acute pain, is vital for the survival of individuals. Pathological pain is caused by tissue damage and can lead to prolonged changes in the periphery and center, leading to hyperalgesia and touch-induced pain [5]. Several chronic pains result from acute pain and their mechanisms are unknown. Pain that usually lasts more than 3 months (or more than 50% of days within 6 months), exceeds the expected healing time, and lacks a critical warning function of physiological nociception is considered chronic [6]. The prevalence of chronic pain is approximately 12%–30%, which severely affects patients' quality of life and results in a substantial socioeconomic burden [7]. Patients with chronic pain usually experience pain with varying degrees of mental and emotional disorders, such as depression and anxiety. Conversely, affective and cognitive disorders are also early risk factors for chronic pain [8,9]. When an injury occurs, pain acts as a warning stimulus to produce a series of defensive responses to protect the body from damage. However, if the pain is severe or persistent, it causes intolerable mental torture to the body [10]. Depression and anxiety represent the most abnormal emotional manifestations associated with pain [11]. In the absence of any antidepressant medication, the depression scores of patients with pain associated with depression also improved significantly as the degree of pain decreased, indicating that patients with pain are often associated with depression and that the severity of depression is related to the degree of pain [12]. In patients with chronic pain, severe impairment of individual functional status and quality of life may induce anxiety. For example, the incidence rate of anxiety in patients with chronic neck and shoulder pain is 35.83%. The degree of anxiety is related to the depression level, duration of disease, body mass index, family relationships, and other factors [13]. In addition, anxiety can also cause pain to persist or worsen. For example, patients who are anxious often do not dare to move because they worry about pain and injury, resulting in impaired physical function and reduced the quality of life.

Currently, clinical practice relieves acute pain by regulating nociceptor afferents, and good results are obtained [14]. However, the treatment of chronic pain remains insufficient. The main reason is that the existing treatment methods cannot effectively relieve patients' psychological and emotional problems, that is, the aversion associated with chronic pain. Patients with chronic pain usually have a strong motivation for pain relief. Recent neuroimaging studies have also shown that adaptive changes in the brain reward/motivation loop may mediate chronic pain's psychological and emotional mechanisms [15]. Thus, the presence of negative emotions of pain, on the one hand, drives an individual to escape from harm in acute pain conditions, and on the other hand, causes the individual to seek relief in chronic pain conditions [16]. Therefore, the goal of pain treatment has also changed from simple pain relief to simultaneous pain reduction behavior, changing patients' negative perceptions of their pain and enhancing self-confidence and self-control [17]. Acupuncture can effectively relieve pain itself and improve cognitive impairment and negative emotions and other complications [18]. This article reviewed the literature on the pain and reward/motivation loop and the effect of acupuncture on this loop to provide new ideas for the internal mechanism of acupuncture exerting multidimensional regulation to produce an analgesic effect.

## 3. Reward/Motivation Circuits

The midbrain limbic reward network, also known as the mesolimbic dopamine (DA) system, is the core region of the reward mechanism [19]. Traditionally, the DA circuit from the ventral tegmental area (VTA) to the nucleus accumbens (NAc) constitutes the basic midbrain reward system. However, in recent years, there has been a new understanding of the DA theory of the central reward system, which shows that the reward loop exists in a complex parallel network [20]. The primary neural basis of the mesencephalic limbic reward network is that the NAc receives neural DA projections from the VTA of the midbrain (i.e., the VTA-NAc pathway), and DA is an essential neurotransmitter in the reward mechanism [21]. In addition, NAc also receives projections from brain regions related to the “emotional” dimension of pain, such as the amygdala (AMY), anterior cingulate cortex (ACC), and prefrontal cortex (PFC), and participates in the coding of reward mechanisms [22–27]. The NAc does not directly encode sensory pain information but can transmit significant signals of emotional values and stimuli [28]. It receives afferent nociceptive information through the projections of the accessory cortex and cortical regions and participates in pain processing. Simultaneously, spinal dorsal horn neurons can also project directly to the NAc. Neurons in the spinal dorsal horn can also project directly to the NAc [29]. In addition, signals transmitted down from the NAc can also modulate spinal nociceptive signals through the medulla's rostral ventromedial medulla region [30]. A previous study [31] used rapid scanning voltammetry to detect real-time DA concentrations and found that aversive stimulation increased DA concentrations in the striatum. The nociceptive tail can induce DA release in the dorsal striatum and NAc core, but in the NAc shell, DA release occurs only after stimulation is terminated. According to the time course of DA activity of NAc shell, it is consistent with the view that pain relief is a reward. A blood oxygen level study that has relied on blood oxygen level dependent-functional magnetic resonance imaging (BOLD-fMRI) measurements has also shown similar results to neurochemical reactions; the NAc shows a negative signal at the beginning of pain but a positive sign at the time of pain relief [32].

**Figure 1:**
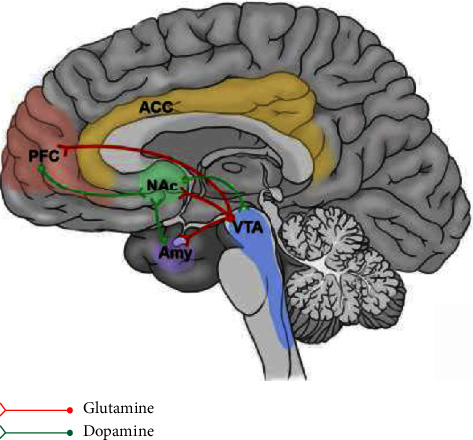
Reward/motivation circuits.

In the brain reward network, the PFC, ACC, VTA, NAc, AMY, and lateral hypothalamus constitute important network nodes that regulate pain and reward. The down-projection circuit of glutamatergic nerves in the PFC forms a critical circuit of cerebral cortex reward [33]. The lateral PFC provides direct or indirect glutamatergic neural projections to the VTA, activates VTA DA neurons, releases DA, and produces reward-related behaviors [34]. Moreover, the PFC, as an essential component of the “pain matrix,” is a critical brain region that regulates pain emotions and participates in pain-related reward and cognition functions [35,36]. Enhancement of neuronal activity in the reward circuit associated with the PFC is a common feature in the formation of chronic pain relief and a core structure of the pain-related brain reward or reinforcement system [37]. Glutamate (Glu) is the most crucial neurotransmitter in the PFC, and its excitatory synaptic transmission is closely related to PFC function. As ionotropic Glu receptors, N-methyl-D-aspartate (NMDA) receptors participate in various crucial nervous system functions, including learning and memory and reward. The NMDA receptor mediates excitatory postsynaptic potentials via the protein kinase C signaling pathway and is a significant regulator of neural synaptic plasticity. A prior study [38] showed that neurons in the PFC underwent long-term plasticity changes during chronic pain. During neuralgia, the PFC reorganizes morphologically and functionally, including Glu-mediated projection circuits and DNA methylation, which are positively correlated with pain sensitivity and mood abnormalities [39] (Figure 1).

## 4. Effect of Pain and Pain Relief on the Reward/Motivation Circuits

Negative emotions produced by pain are an important part of pain perception. However, the reward mechanism is the self-regulation mechanism of the body's nervous system to relieve negative emotions and promote positive emotional states [22]. In a recent study on chronic pain, the elimination of negative emotions and the return to a normal state are used as indicators of pain relief. A prior psychological study [40] has suggested that properly handling a state of disgust is rewarding. Although the idea that pain relief can be a reward has long appeared in human studies [41,42], it has only recently been demonstrated in animals [43,44]. Traditional animal models have limitations in exploring pain by measuring the pain threshold that does not truly reflect animal pain emotions [45]. In animal models, the conditioned position paradigm is often used to determine the emotional dimension of animal pain. The paradigm uses a specific position or environmental signal as a conditioned stimulus and a reward or punitive stimulus as an unconditioned stimulus. The two are repeatedly paired and reinforced to form a conditioned reflex. The animal's exposure to the environment or signal will show a clear preference or escape response. Negative emotions in animals show conditioned place aversion (CPA) when pain is associated with environmental cues [46].

Conversely, chronic pain's palliative or analgesic effect produces a sense of pleasure that manifests as a positive emotional state or as a negative reinforcement of pain, which is considered a reward that activates the brain reward system [22,47]. The administration of analgesic and non-analgesic drugs to slow pain model animals resulted in conditioned place preference (CPP). This behavior of CPP triggered by pain relief is closely related to the activation of mesolimbic circuits. Some researchers used peripheral nerve blockade to relieve persistent pain in rats [43]. After pain relief, the expression of DA neurons in the VTA region was significantly increased, and the release of DA in the NAc shell increased considerably. If the expression of DA neurons or the transmission of DA signals was inhibited, the occurrence of CCP behavior could be hindered. Notably, pain relief also significantly affects the midbrain limbic reward network [48]. Researchers [49] used the CPA model to study the negative emotions of pain. During training, rats were injected with formalin, a pain-causing substance, in box A, and no drug was administered in box B. During the test phase, the animals were allowed to select their stay position without freely being in painful stimulation. The results showed that the rats had significantly shorter stay time in box A; that is, they showed avoidance of box A. This suggests that the formalin-induced CPA (F-CPA) model is successfully developed, reflecting the aversion associated with spontaneous formalin-induced pain. ACC receives nociceptive signals from the medial thalamus through the spinal-thalamic pathway, and its rostral portion (rostral anterior cingulated cortex (rACC)) plays an important role in the processing of pain aversion. Microinjection of excitatory amino acids into rACC produces CPA without affecting the sensory threshold [50]. In contrast, destruction of rACC attenuates F-CPA but does not alter the pain response induced by formalin alone. The inhibition of rACC extracellular signal-regulated kinase activation blocks F-CPA expression [51].

Pain and pain relief can significantly influence reward/motivational circuits [52]. BOLD-fMRI activation is typically observed in reward/motivation networks, including the orbitofrontal cortex, NAc, and VTA, following acute noxious stimulation [53]. A previous study [54] using positron emission tomography (PET) imaging to visualize DA receptors showed that nociceptive stimulation increased DA neurotransmission in NAc. In addition, this pain-induced dopaminergic activation within NAc is associated with changes in individual negative emotional evaluation during pain. Another PET scan study found that the D2/D3 receptor binding potential in the ventral striatum of patients with chronic nonneuropathic back pain was lower compared with that of healthy subjects. The D2/D3 receptor binding potential in this brain region was negatively correlated with the positive affective state score and pain tolerance evaluation in patients with chronic back pain [55]. Animal experiments have also observed that CPP after sustained pain relief is closely related to the activation of DA energy in the mesolimbic circuit. Peripheral nerve blockade relieves persistent postoperative pain in rats, which can significantly induce CPP behavior in animals. Meanwhile, the expression of Fos in DA neurons in the VTA region is increased substantially, and the release of DA in the NAc shell is also significantly increased. However, inhibiting VTA pain relief-induced CPP can be blocked by blocking DA signaling in regional DA neurons or by blocking DA signaling within the NAc, providing direct evidence for the association between pain-motivational behavior and midbrain reward/motivation circuits. In addition, in the rat head pain model, pain relief can activate DA neurotransmission in NAc [56]. The abovementioned results suggest that the activation of the midbrain reward/motivation circuit can reflect the degree of pain relief so that the analgesic effect of a particular therapeutic measure can be indirectly judged by observing the activation of this circuit [57].

Therefore, the midbrain reward system plays a crucial role in the perception and regulation of chronic pain symptoms. Chronic pain stimulation changes the functional structure of the midbrain limbic reward network, and the release of neurotransmitters is significantly reduced, leading to impairment of the reward effect, which leads to or aggravates chronic pain. Moreover, the activation of the midbrain limbic reward mechanism can reflect the degree of pain relief; that is, the midbrain limbic reward network is closely related to chronic pain [58], and the reward effect of pain relief is closely associated with the activation of the brain reward system [43,59–64].

## 5. Effects of Acupuncture on the Brain Reward/Motivation Circuits

Acupuncture, as a technique of peripheral nerve regulation, has paid significant attention to the role of the central circuit [16]. Previous studies have pointed out that acupuncture analgesia can activate the brain reward circuit [16,65–67]. A previous study [68] has reported that the analgesic effect of acupuncture affects the “self-evaluation” of the human body and activates the brain reward system, with the activation of the brain striatal system being an essential mechanism for acupuncture to produce pleasure [69]. Functional nuclear magnetic resonance studies [70–72] have shown that acupuncture produces an analgesic effect by modulating the limbic lobe-limbiclobe-neocortex network system. That is, acupuncture can modulate the activity of the limbic cortex and subcortical structures, including the PFC, anterior cingulate gyrus, AMY, hippocampus, and hypothalamus. A prior study [73] used fMRI to study the effects of acupuncture with different cues on brain activation. Participants in the “acupuncture treatment group” were informed that acupuncture was part of the treatment, whereas participants in the “acupuncture stimulation group” were informed that acupuncture was a pain stimulus. The two groups received the same amount of acupuncture, and there was only a difference in language guidance. The results of the study showed that both groups of acupuncture could activate brain regions (ventral striatum) related to brain reward, but the “acupuncture treatment group” produced more robust brain activation signals than the “acupuncture stimulation group.” Another fMRI study [74] showed that improving patients' expectation of acupuncture enhanced the therapeutic effect of knee osteoarthritis pain, and the patient's expectation score was positively correlated with the reduction of pain and activation degree of related brain regions (NAc, anterior cingulate gyrus, ventral PFC). Using resting fMRI, acupuncture treatment of chronic low back pain can also activate the brain reward system, significantly enhancing the functional connection of the VTA/aqueductal gray to the AMY circuit [65]. An fMRI study based on a mixed-effects model showed that immediate relief of chronic pain and negative emotions by acupuncture were associated with significant changes in spontaneous neural activity in the default network, basal ganglia region, and brain region where the limbic system is located, and NAc, especially in contrast to the spontaneous neural activity of chronic pain in the insular and bilateral occipital lobes [75]. Notably, a previous study [76] has shown that acupuncture improves depressive symptoms in patients with severe depression and activates the brain reward circuit. Considering that acupuncture also activates the reward loop in the brain of healthy subjects, these results suggest that the reward loop in the brain is a standard mechanism by which acupuncture modulates emotional disorders. In addition, animal experiments also indicate the modulation of the reward system by acupuncture. For example, acupuncture regulates opioid peptide receptors in the NAc, Glu, and gamma-aminobutyric acid in the AMY, and pyramidal neurons in the anterior cingulate gyrus [77]. A prior study [78] has shown that electroacupuncture relieves emotional pain in an animal model of inflammatory pain involving the anterior cortical opioid peptide system. The effects of acupuncture on glutamatergic pyramidal neurons in the ventral medial PFC were observed by multichannel electrophysiology and immunohistochemistry [79]. A recent study [80] has also highlighted the importance of cortical mechanisms, reporting that the effect of electroacupuncture on pain relief may be achieved through cortical loop regulation mediated by cannabinoid receptor 1. Pariente et al. [81] used PET scanning to study the effect of patients' expectations and beliefs on acupuncture analgesia. The results showed that the honest acupuncture and comfort acupuncture (with the same treatment expectation) groups had more robust activation in the midbrain, right dorsolateral PFC, and ACC than the skin prick group (with the expectation of treatment effect).

In addition, acupuncture can also regulate several neurochemicals in the brain reward/motivation system. Electroacupuncture promotes the release of opioids from the central nervous system to exert its analgesic effect. After the activity of opioid receptors in the NAc is blocked by opioid receptor antagonists, the analgesic effect of acupuncture is reduced [82,83], and rACC endogenous opioid signals are necessary for relieving pain aversion [64,78]. In addition, electroacupuncture can activate the brain reward system and increase the 5-hydroxytryptamine (5-HT) level in the NAc of bound conscious rats, suggesting that acupuncture may be effective in the treatment of emotional disorders [84]. Recent studies have shown that repeated electroacupuncture has a pronounced analgesic effect on rats with chronic neuralgia and can significantly improve their pain aversion. The effect of electroacupuncture on pain aversion may be related to the upregulation of GluAl, metabotropic glutamate receptor 1 (mGluR1), *γ*-aminobutyric acid (GABA)B2 protein, and Piccolo gene expression in AMY [85,86]. Acupuncture can relieve pain and negative emotion simultaneously as analgesia. An fMRI study showed [74] a positive correlation between the efficacy of acupuncture in treating knee osteoarthritis pain and the degree of activation of brain regions related to the limbic reward system (NAc, PFC, and ACC). The study also proposed that the degree of pain relief and activation of related brain regions would be increased when patients received the same treatment. Similar to another study, acupuncture with different cues was found to increase the DA level in the relevant nuclei in the reward network, thus inhibiting pain [73,87].

The series suggested the widespread effects of acupuncture on the brain reward system. Notably, if the extensive influence of acupuncture on the brain reward system reflects the broad spectrum of acupuncture effects [73], then the specific core reward loop of acupuncture analgesia reward has yet to be clarified. Optogenetic techniques provide a crucial research tool for the detailed study of cerebral circuits in acupuncture analgesia [88]. The expression of light-sensitive proteins in a specific cell population of a single source (excitatory or inhibitory neurons or glial cells) by genetic engineering methods so that cells that do not express light-sensitive proteins does not respond to light stimulation, and the use of light to regulate neurons noninvasively and reversibly has high accuracy in studying the regulation of specific cell populations in the reward circuit.

In particular, the new generation of optogenetic technology combines the supermicro calcium imaging system of free-moving neurons with the optogenetic supermicro calcium imaging system of free-moving neurons, which has the characteristics of cell-specific regulation, high-precisionspatial-temporal resolution, reproducibility, and functional anatomy of neural networks. It achieves the high temporal, spatial, and cell resolution required for acupuncture studies. If it is combined with nervous electrophysiology and integrated with behavioral data, it dramatically enriched the experimental contents and results and made the study results more convincing [89,90].

## 6. Summary and Outlook

In summary, because patients with chronic pain experience pain and negative emotions, pain relief needs to not only alleviate the physiological aspect of pain but also return negative emotions (pain aversion) to normal as a reference index. Previous studies have demonstrated that adaptive changes in the brain reward/motivation loop during chronic pain may be involved in and worsen the emotional response to chronic pain. Acupuncture, including electroacupuncture, can significantly inhibit pain sensation with the immediate analgesic effect and improve pain-induced negative emotions, including anxiety and depression accompanied by pain and cognitive changes. The brain reward motivation system is activated simultaneously as acupuncture analgesia, which can relieve pain aversion by promoting the release of opioids and 5-HT in the brain reward/motivation circuit and upregulating the expression of AMY's (Glu)Al, mGluR1, and GABAB2 proteins, thus exerting its analgesic effect.

Several problems need to be solved in the reward effect of acupuncture analgesia. First, a previous study [22] has shown that the pain sensitivity of rats reaches a peak 24 h after surgical incision pain. The pain still exists 96 h after the operation, but the pain sensitivity is significantly reduced. The experimental animals produced significant CPP by nerve block with lidocaine 24 h after the incision. However, after 96 h of modeling, the nerve block with lidocaine could not produce CPP, suggesting that the course of the disease affects the reward effect. Does the reward effect of acupuncture analgesia also have the characteristic of aging? Especially for chronic pain, the course of the disease is often long, and the central nervous system undergoes plasticity changes. Second, whether different acupuncture treatment schemes, such as acupuncture frequency, manipulation, selection of acupuncture points, and different electroacupuncture frequencies and current intensities, have other effects on analgesic reward effect remains to be further studied. Third, the formation of the reward effect of acupuncture analgesia itself is a type of conditional learning process that involves the coding, consolidation, and reconsolidation of learning and memory. We must deeply understand the similarities and differences between the reward learning process of acupuncture analgesia and the standard positive emotion learning process, which will provide necessary mechanism support for acupuncture treatment of patients with chronic pain.

It is of great significance to study the reward effect of acupuncture in relieving chronic pain. First, many patients with chronic pain expect the pleasure of acupuncture to relieve pain. However, it is unclear whether this is the motivation for patients to seek further acupuncture treatment. This is an important clinical question to be answered in the basic study of acupuncture. Second, the characteristics of pain reward and its regulation mechanism are essential manifestations of an organism to maintain homeostasis in the face of environmental challenges. The idea of controlling chronic pain by changing the reward/motivation system is of great concern. Third, the reward effect of pain relief will drive the motivation and behavior related to pain relief, thus accelerating the pain relief, which is of great clinical significance. Finally, studies have shown that significant alterations in pain relief reward are closely related to analgesic tolerance. Therefore, if we can study the reward effect of acupuncture on pain relief (negative enhancement of pain) or the regulating effect of the brain reward system on acupuncture analgesia, we will innovate the study concept of acupuncture analgesia and provide new ideas and perspectives for further elucidating the mechanism of acupuncture analgesia. Given the association between pain and the brain reward/motivation system, neuroimaging technology can be used to trace brain regions that mediate acupuncture action. The response of the brain reward/motivation system can be used as an objective indicator to determine the analgesic efficacy of acupuncture, which will provide help for further exploring the central mechanism of acupuncture to improve chronic pain in the future.

## Data Availability

The data used to support the findings of this study are included within the article.
